# The 626M24 dataset of validated transitions and empirical rovibrational energy levels of ^16^O^12^C^16^O

**DOI:** 10.1038/s41597-025-04755-w

**Published:** 2025-03-29

**Authors:** Ala’a A. A. Azzam, Dunia Alatoom, Bashar M. J. Abou Doud, Meera Q. A. Shersheer, Bailasan K. M. Almasri, Celin N. M. Bader, Baraa O. A. Kh. Musleh, Maria Zakaria Jado Obaido, Ahmad M. H. Abu Khudair, Adam W. M. Al Shatarat, Bana I. M. Qattan, Loay H. M. Hamamsy, Abdullah O. G. Saafneh, Mohammad N. A. ALso’ub, Mera M. A. Alkhashashneh, Haneen O. M. Al-Zawahra, Mohammad Taha I. Ibrahim, Jonathan Tennyson, Sergei N. Yurchenko, Tibor Furtenbacher, Attila G. Császár

**Affiliations:** 1 Department of Physics, The University of Jordan, Queen Rania St, Amman, Jordan; 2 Research Department, AstroJo Institute, Wasfi Al-Tal St, Amman, Jordan; 3 Department of Physics and Astronomy, University College London, Gower Street, London, WC1E 6BT UK; 4 Pioneer Educational Schools, Dhi Al Qaada, Amman, Jordan; 5 Bunat AlGhad Academy, Shaher Abu Haya, Aws Ben Thabet St., Amman, Jordan; 6 First University Schools, Jubaihah, Ahmad Altarawneh St., Amman, Jordan; 7 International Grand Academy, GW3J+6VQ Petra St., Aydoun, Jordan; 8 Islamic Educational College, 2V8R+GV6 Al-Hakem An-Nisabouri St., Amman, Jordan; 9 Modern Systems Schools, Tla’a Al Ali, Amman, Jordan; 10 AlJami’a AlOula Schools, Ahmad Al-Tarawneh 65, Amman, Jordan; 11 Mada International Academy, King Abdullah II St. 379, Amman, Jordan; 12 The Jubilee School, 2WJ2+FVH Muhammad An-Nejdawi St., Amman, Jordan; 13 AlHassad AlTarbawi Schools, VVPW+X6M At-Tahrir St., Amman, Jordan; 14 ELTE Eötvös Loránd University, Institute of Chemistry, H-1117 Budapest, Pázmány Péter sétány 1/A, Hungary

**Keywords:** Atmospheric chemistry, Atmospheric chemistry

## Abstract

The 626M24 dataset created during this project contains validated experimental transitions and empirical rovibrational energy levels for the parent carbon dioxide isotopologue, ^12^C^16^O_2_ (in a shorthand notation, 626). Validation of the measured transitions and determination of the empirical energy levels is based on a compiled and carefully checked dataset of experimental rovibrational transitions collected from 143 literature sources. The 44 828 measured lines collected, in the wavenumber range of 42.9 – 14 076 cm^−1^, describe 22 218 unique transitions. Inversion of the experimental information yields 8268 empirical rovibrational energy levels for 626, with uncertainty estimates compliant with the experimental uncertainties of the transitions. Comparison with the Carbon Dioxide Spectroscopic Databank (CDSD-296), NASA Ames-2021, and HITRAN2020 line catalogues shows generally good agreement and suggests some possible improvements to these databases. The 626M24 dataset and an extended line list, called 626M24LL, built upon it and containing 285 503 line positions, are deposited in an OSF (Open Science Framework) repository.

## Background & Summary

The triatomic carbon dioxide molecule, CO_2_, contains two terminal oxygen and a single central carbon atom, and in the electronic ground state it has a linear equilibrium structure. Studying the internal motions and related rovibrational spectra of carbon dioxide at relatively low temperatures, so that none of the excited electronic states need to be taken into account, is relevant to many fields of science and technology.

The variation in the CO_2_ content of the atmosphere of Earth over time is one of its important characteristics^1^. Detailed understanding of CO_2_ spectroscopic features^2,3^, in particular the line center positions, both with and without collisional effects, helps to establish the total amount and the distribution of this molecule in the atmosphere of Earth. These data also help to understand the effect of CO_2_ on a number of environmental issues, such as climate change and the radiative balance of our atmosphere. The industrial revolution has had a significant impact on climate change; during the last century, the well-documented increase of CO_2_’s concentration in the atmosphere of the Earth is predominantly due to human activity^4^. Approximately 98.45 % of carbon dioxide molecules in the atmosphere of the Earth are in the form of the parent, ^16^O^12^C^16^O isotopologue, making it the most important isotopologue to study.

Carbon dioxide plays a crucial role in many areas of astronomical research as well, from the study of stars^5,6^ to the exploration of planetary atmospheres^7–9^: in our own solar system, carbon dioxide is the major constituent and thus determines the radiative balance of the atmospheres of the planets Mars and Venus. The detection and characterization of CO_2_ absorption features in exoplanetary spectra offer clues about the atmospheric composition, pressure, and temperature profiles of these distant worlds. CO_2_ was one of the first molecules detected in the atmosphere of an exoplanet^10^ and several recent observations have been made using the James Webb Space Telescope^11^. Within the dense molecular clouds that pervade the interstellar medium, CO_2_ serves as an important tracer of the physical and chemical conditions that govern the birth of new stars^12,13^. Emissions from the bending states of the CO_2_ isotopologues in the far infrared provide valuable information on the temperature, density, and kinematics of these star-forming regions^12^.

Remote sensing of the CO_2_ content of the Earth’s atmosphere is a major activity aimed at monitoring the carbon content of our atmosphere in increasing detail. Missions such as NASA’s OCO-2 and OCO-3 satellites and ESA’s planned CO2M satellite constellation have stringent requirements on laboratory spectroscopy results, required for the interpretation of their observations^14,15^. Similar accuracy is required for ground-based spectroscopic experiments such as TCCON (Total Carbon Column Observing Network)^16^.

CO_2_ spectra are important for medical^17^ and industrial applications^18^, as well. The study of CO_2_ spectra in plasma physics is widespread^19^, where there is particular emphasis on the use of plasma processes to valorize excess CO_2_ from the Earth’s atmosphere^20^.

Due to the importance of accurate high-resolution spectroscopic data related to carbon dioxide, they are available in several line-by-line spectroscopic databases, such as the Carbon Dioxide Spectroscopic Databank (CDSD-296)^21^, NASA Ames-2021^22^, HITRAN2020^23^, and ExoMol^24^. The present report on the spectroscopic data of ^16^O^12^C^16^O (626) is part of a long-term, ongoing project devoted to the construction of the most extensive empirical energy level datasets, calculated from measured line positions in high-resolution rovibrational spectra, for all isotopologues of carbon dioxide involving the ^12^C, ^13^C, ^16^O, ^17^O, and ^18^O isotopes. Empirical energy levels based on *all* the measured rovibrational transitions are already available for the carbon dioxide isotopologues ^16^O^12^C^18^O (628)^25^, ^16^O^13^C^16^O (636)^26^, ^16^O^13^C^18^O (638)^27^, ^18^O^12^C^18^O (828)^28^, ^17^O^12^C^18^O (728)^28^, and ^18^O^13^C^18^O (838)^28^ (see Table 1). In these projects, empirical energy levels are calculated using the MARVEL 4.0 (Measured Active Rotational-Vibrational Energy Levels) procedure^29–32^, built upon the theory of spectroscopic networks^33,34^. Statistical measures of these previous studies, mostly with respect to the Ames-2021^22^ and CDSD-296^21^ datasets of energy levels are also given in Table 1. For the seven isotopologues studied thus far, agreement with the CDSD-296 data is significantly better, by more than an order of magnitude for the average absolute deviation.

**Table 1:** The number of empirical (MARVEL) rovibrational energy levels determined, and the maximum absolute (MAD) and average absolute (AAD) energy level differences, *ΔE*, in *hc* cm^−1^, between the MARVEL studies and those from Ames-2021^22^ and CDSD-296^21^ for isotopologues of carbon dioxide studied by our group^25– 28^.

Isotopologue	No. of levels	${\boldsymbol{\Delta }}{{\text{E}}}_{{\bf{A}}{\bf{m}}{\bf{e}}{\bf{s}}-{\bf{2021}}}^{{\bf{M}}{\bf{A}}{\bf{D}}}$	${\boldsymbol{\Delta }}{{\text{E}}}_{{\bf{A}}{\bf{m}}{\bf{e}}{\bf{s}}-{\bf{2021}}}^{{\bf{A}}{\bf{A}}{\bf{D}}}$	${\boldsymbol{\Delta }}{{\text{E}}}_{{\bf{C}}{\bf{D}}{\bf{S}}{\bf{D}}-{\bf{296}}}^{{\bf{M}}{\bf{A}}{\bf{D}}}$	${\boldsymbol{\Delta }}{{\text{E}}}_{{\bf{C}}{\bf{D}}{\bf{S}}{\bf{D}}-{\bf{296}}}^{{\bf{A}}{\bf{A}}{\bf{D}}}$
^16^O^12^C^16^O (626) [This work]	8268	0.147	0.015	0.077	0.001
^16^O^12^C^18^O (628)^25^	8786	0.231	0.022	0.182	0.002
^16^O^13^C^16^O (636)^26^	6318	0.152	0.016	0.081	0.002
^16^O^13^C^18^O (638)^27^	3975	0.151	0.024	0.268	0.002
^18^O^12^C^18^O (828)^28^	3923	0.571	0.042	0.559	0.002
^17^O^12^C^18^O (728)^28^	4318	0.109	0.024	0.046	0.001
^18^O^13^C^18^O (838)^28^	1058	0.168	0.045	0.039	0.001

The most important results of this study, obtained with the help of the MARVEL code for the 626 isotopologue of carbon dioxide, include the 626M24 dataset of validated experimental transitions and empirical rovibrational energy levels, and a large rovibrational line list, 626M24LL. All of these data will contribute not only to future spectroscopic measurements on carbon dioxide but also to the refinement of theoretical and computational spectroscopic models and the enhancement of spectroscopic line-by-line databases, such as HITRAN^23^ and ExoMol^24,35^.

## Methods

### Source data

References^36–180^ contain rovibrational transitions data considered during the MARVEL analysis of this study. The wavenumber range covered by these measurements is limited to 42.9 – 14 076 cm^−1^.

### MARVEL

The MARVEL procedure^29–32^, used extensively during this study, starts with the careful collection, detailed examination, and subsequent validation of the positions of transitions in high-resolution (laboratory) spectra. The transitions collected are then used to construct a spectroscopic network (SN)^33,34^, whereby each energy level serves as a node and the nodes are interconnected by the observed transitions. The SN built allows the determination of empirical energy-level values along with educated estimates for their uncertainties^32^. Unlike the effective Hamiltonians widely used for spectroscopic analysis, the MARVEL approach is model-free. This has a number of advantages and, in particular, for the CO_2_ molecule with its many resonances, MARVEL does not require any special measures or extra parameters to characterize levels perturbed by “accidental” interactions with nearby states.

Ideally, the experimentally observed transitions allow the creation of a well-connected SN, linking all transitions to the ground state (defined as the state with no rovibrational excitation), called the root of the SN. However, because of the limited coverage offered by the experimental data, this is usually not the case. Therefore, in practice, the SN can become fragmented, resulting in a principal component, where all the nodes are linked to the root, and a number of isolated, so-called floating components with their own roots.

The MARVEL protocol allows for the detection of inconsistencies, that is, lines that are in conflict with the correct measurement data. This feature proves invaluable for identifying issues with experimental data that usually come from several sources, such as user errors made during data collection and analysis, incorrect assignments, or the use of different naming conventions.

### Notation and quantum numbers

CO_2_ has three fundamental vibrational modes, conventionally denoted as *ν*_1_, *ν*_2_, and *ν*_3_, associated with the vibrational quantum numbers *v*_*i*_, *i* = 1, 2, and 3, respectively. The two-dimensional (degenerate) bending mode, *ν*_2_, is characterized by an angular momentum, described by the quantum number *ℓ*_2_. Herzberg’s notation is often used to assign energy levels in triatomics; in this notation, the vibrational states of CO_2_ are designated as $({v}_{1}\,{v}_{2}^{{\ell }_{2}}\,{v}_{3})$. For the CO_2_ molecule with a linear equilibrium structure in its ground electronic state, there is a strong Fermi-resonance interaction between the states (${v}_{1}\,{({v}_{2}+2)}^{{\ell }_{2}}\,{v}_{3}$) and (${v}_{1}+1\,{v}_{2}^{{\ell }_{2}}\,{v}_{3}$). Therefore, it became customary to employ the so-called AFGL (Air Force Geophysics Laboratory) notation to denote the vibrational states and bands of CO_2_ isotopologues. In the AFGL notation^181–183^, the vibrational energy levels are designated by the quintuplet (*v*_1_ *v*_2_ *ℓ*_2_ *v*_3_ *r*), where *r* is the ranking index for states in Fermi resonance (the *r* index is used to distinguish the levels belonging to the same Fermi polyad). The lowest value of *r*, 1, is assigned to the energy level with the highest wavenumber (or frequency), and *r* increases for lower-energy levels. For example, the three vibrational states (2 0^0^ 0), (1 2^0^ 0), and (0 4^0^ 0) are in Fermi resonance with each other and have the AFGL vibrational descriptors (2 0 0 0 3), (2 0 0 0 2), and (2 0 0 0 1), respectively.

It is customary to use polyad numbers *P* to denote strongly interacting groups of vibrational states, decoupling them from the other vibrations. This is a useful concept, especially when effective Hamiltonians are formed. *P* is not a quantum number, but it behaves like one. For carbon dioxide, based on the approximate relations of the harmonic frequencies, *ω*_1_ ≈ 2*ω*_2_ and *ω*_3_ ≈ 3*ω*_2_, the widely accepted definition of *P*, also used in this study, is *P* = 2*v*_1_ + *v*_2_ + 3*v*_3_.

The quantum number *J* is used to denote the angular momentum associated with rotational and (when *ℓ*_2_ > 0) vibrational motion of the CO_2_ molecule. Transitions with *ΔJ* = − 1 and *ΔJ* = + 1 are called the P- and R-branch transitions, respectively, while the Q-branch transitions are associated with *ΔJ* = 0. P and R transitions occur in both the parallel and perpendicular bands, while the Q branch transitions only occur in the parallel bands, where the direction refers to the change in the dipole moment driving the transition relative to the linear equilibrium structure of the molecule. For the symmetric isotopologue 626, the Pauli principle means that symmetric vibrational states (those with even *v*_3_ values) only have even *J* levels, while anti-symmetric vibrational states (those with odd *v*_3_ values) have only odd *J* levels. Similarly, for states with even values of *J* + *ℓ*_2_ + *v*_3_ the rotationless parity is ‘e’, while for states with odd *J* + *ℓ*_2_ + *v*_3_ values the rotationless parity is ‘f’. The coupling of rotational and vibrational angular momentum means that *J* ≥ *l*_2_.

The upper and lower states involved in a transition are denoted as ′ and *″*, respectively, and the P, R, and Q transitions are usually specified using the lower-state rotational quantum number (*J*″). For the purposes of the MARVEL analysis, each state is uniquely characterized using the set of seven descriptors (*J* *v*_1_ *v*_2_ *ℓ*_2_ *v*_3_ *r* *e*/*f*). This is the format followed by the data deposited in the Supplementary Material to this paper.

## Data Records

The 626M24 dataset is available in an OSF (Open Science Framework) repository^184^. It contains (a) all experimentally measured transitions collected during this work, (b) all empirical rovibrational energy levels determined, and (c) an extensive line list derived from the levels. All validated transitions have positive wavenumber or frequency values, while transitions that had to be removed have negative values. The same repository contains a table describing the main characteristics of the 143 literature sources that contain the transitions collected and analyzed.

The file “626M24_segments.txt” is the segment input file utilized by the MARVEL code, where the unit of the line positions and their uncertainties are specified for each data source. The file “626M24_transitions.txt” contains the 44 828 input transitions, collected from Refs. ^36–180^, used during the MARVEL analysis. In this file, each transition is characterized by (a) a line position (in units stored in the segment file), (b) an initial and an adjusted line-position uncertainty, (c) the rovibrational assignments for the upper and lower states (see the previous section for a description), and (d) a line tag, representing a unique identifier (each data source tag is based on the last two digits of the year of publication and the first two characters of the last names of the authors).

Of all the experimentally measured transitions only about half of them, 22 218, are unique. During the MARVEL analysis, 368 transitions had to be removed from our spectroscopic network; note, in particular, that all the measured transitions of 00TaPeTeLe^107^ had to be deleted. It is also worth mentioning that although the transitions of the sources 94Bailly^93^ and 97BaCaLa^97^ are included in the transition file, the transitions reported in them form floating components. Thus, we cannot independently validate them or determine the absolute values of the energy levels associated with them. Finally, it is important to add that although most of the transitions in the transition file “626M24_transitions.txt” are from measurements, the final dataset also contains calculated line positions. These sources are denoted by ‘_C’ in the tag. The reason to include these calculated line positions in the transitions list is that the uncertainty of these lines is several orders of magnitude smaller than that of other transitions measured in the given region and they help the analysis of the spectroscopic network of 626.

The empirical energy values, obtained for 8268 rovibrational states in the 0 − 20 654 cm^−1^ range, are placed in the file “626M24_energy_levels.txt”. Each energy level of this data file is characterized by (a) a rovibrational label, (b) an empirical (MARVEL) energy in *hc* cm^−1^, (c) an energy uncertainty in *hc* cm^−1^, and (d) the number of transitions incident to this state.

Using our empirical energies and the CDSD-296^21^, NASA Ames-2021^22^, and HITRAN2020^23^ line positions and intensities, an extended line list, named 626M24LL, was constructed, given in the file “626M24_line_list.txt”. Line intensities relate the probability of absorption by a given line at a specified temperature; here we adopt the standard HITRAN^23^ unit of cm molecule^−1^. This line list contains 285 503 dipole-allowed transitions in the range 147 − 19 909 cm^−1^, with room-temperature intensities down to 10^−31^ cm molecule^−1^. Columns (1) − (21) of the “626M24_line_list.txt” file contain the following information: (1) CDSD-296 line position, (2) AMES-21 line position, (3) HITRAN2020 line position, (4) MARVEL line position (generated from the MARVEL energy levels as *E*_up,MARVEL_ − *E*_low,MARVEL_), (5) MARVEL uncertainty, (6) AMES-21 intensity (100% abundance assumed), (7) HITRAN2020 line intensity (scaled by natural abundance), (8-14) descriptors of the upper state, and (15-21) descriptors of the lower state. All line positions and uncertainties are in cm^−1^, the intensity values correspond to a temperature of 296 K. Beyond column (21) the line may contain a possible comment. Four types of comments are used: (a) ‘ONLY IN MARVEL’ means that this line can only be found in the experimental transitions dataset, but not in CDSD-296 and HITRAN2020. There are 290 such lines in the 626M24 dataset, typically with high *v*_3_ values (*v*_3_ > 6). (b) The 626M24 dataset contains 2506 lines that can be found ‘ONLY IN HITRAN’. Most of these lines (2134) are not assigned (their vibrational labels are –2 –2 –2 2 0). (c) When the deviation of the HITRAN2020 position from the CDSD-296 and/or MARVEL positions is larger than 0.01 cm^−1^, the comment ‘Incorrect HITRAN line position’ is used. These 565 HITRAN2020 lines should be reinvestigated and replaced with CDSD-296 or MARVEL positions. (d) When the deviation of the MARVEL position from the CDSD-296 position is larger than 0.005 cm^−1^, the comment ‘Conflict with MARVEL’ is used. There are 5110 such cases. They are divided into two groups. First, when both the lower and the upper energy levels are determined by at least three transitions, *i.e*., the MARVEL prediction is considered to be reliable, ‘!’ is used at the end of the comment. For example, three sources^76,88,101^ measured a line at 3181.915 cm^−1^, but the CDSD-296 position of this line is 3181.909 cm^−1^. Most of the cases (4427 occurrences) belong to the second group, where one of the MARVEL energy levels, typically the upper energy level, is defined by only one or two transitions. In this case, ‘?’ is placed at the end of the comment, denoting that it is possible that the experimentally measured line is not reliable. As a point of interest, note that while the initial dataset, “626M24_transitions.txt”, contains 816 transitions with uncertainties of less than 10^−6^ cm^−1^, the number of such transitions in the extended line list, “626M24_line_list.txt”, is 2101.

Finally, the file “626M24_MARVEL.exe” is a developer version of the MARVEL code, written in the C++ language. This version of the MARVEL code, distributed with the necessary input files ("626M24_transitions.txt” and “626M24_segments.txt”), was used to generate the numerical data of the 626M24 repository.

## Technical Validation

**Fig. 1:**
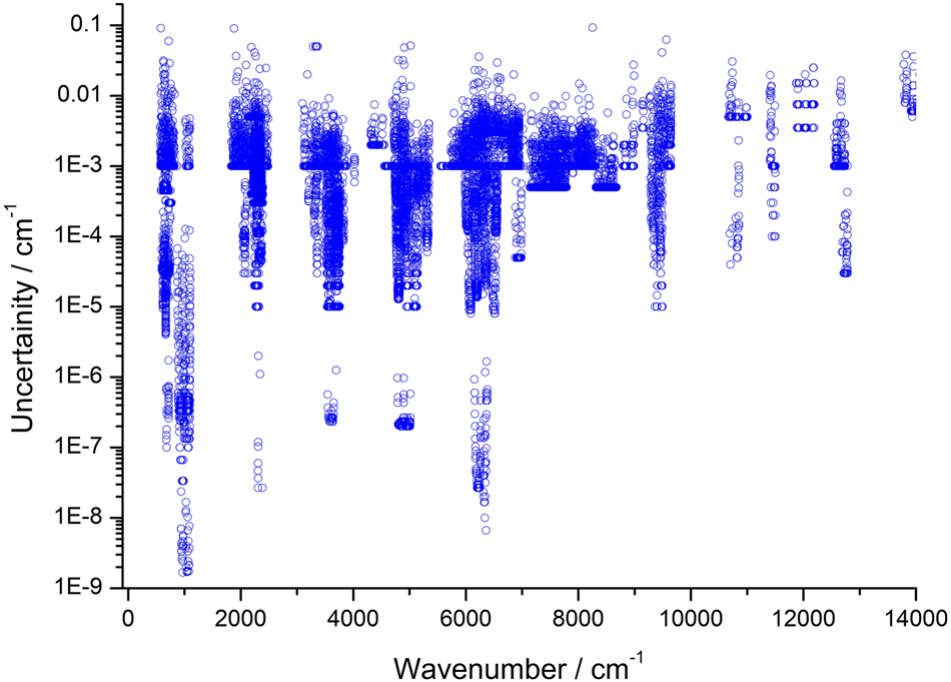
Uncertainties of the experimental rovibrational line-center positions available for the ^16^O^12^C^16^O molecule, as a function of the transition wavenumber (note the logarithmic scale of the vertical axis). If multiple measurements are available for the same line, the most accurate transition is chosen.

The principal validation of the 626M24 energy levels was performed *via* the MARVEL procedure (see Sec. 2.2). Basically, it involved an elaborate checking of the consistency of the experimentally measured transitions collected, in relation to their assignments, line positions, and uncertainties. Figure 1 shows the final experimental uncertainties of the validated rovibrational measurements of ^16^O^12^C^16^O as a function of the transition wavenumber. This figure shows that for ^16^O^12^C^16^O, one of the spectroscopically most studied molecules, (a) the uncertainties of the experimentally measured transitions cover almost eight orders of magnitude, from 1 × 10^−9^ to 10^−1^ cm^−1^, (b) the wavenumber range covered by the experiments is rather limited, only going up to 14 000 cm^−1^, and (c) there are no highly accurate measurements above 7000 cm^−1^.

The global MARVEL analysis resulted in the best rovibrational energy-level dataset, based on the presently available transitions. An important validation of the 626M24 energy values is their comparison with entries in standard databases. Comparison of the predicted line positions of 626M24 with those in the CDSD-296^21^, Ames-2021^22^, and HITRAN2020^23^ line catalogs is particularly important, as it allows additional validation of the energy dataset derived in this study. Furthermore, this comparison might reveal database entries that require further verification and/or modification.

**Fig. 2:**
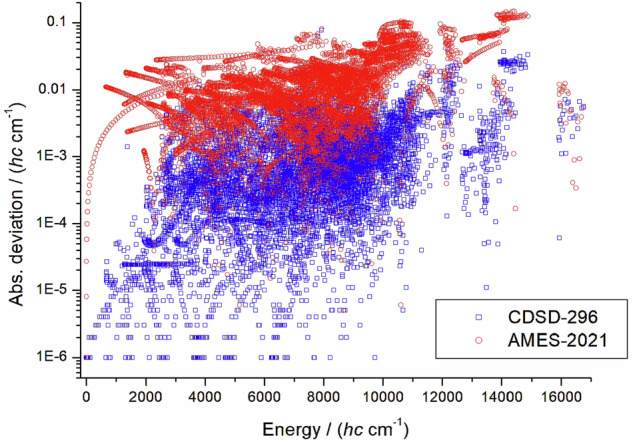
Comparison between rovibrational energies of the present ^16^O^12^C^16^O dataset and those of CDSD-296^21^ (blue squares) and Ames-2021^22^ (red circles).

Figure 2 shows the absolute deviations between the MARVEL data and those of CDSD-296 and Ames-2021. The MARVEL data show significantly better agreement with CDSD-296 (with a root-mean-square, rms, deviation of 0.0032 *hc* cm^−1^) than with Ames-2021 (with an rms value of 0.0238 *hc* cm^−1^, which is an order of magnitude higher). This is not surprising, as the CDSD-296 data are semi-empirical in nature. A comparison with the HITRAN2020 data can be found in the file “626M24_line_list.txt”.

**Fig. 3:**
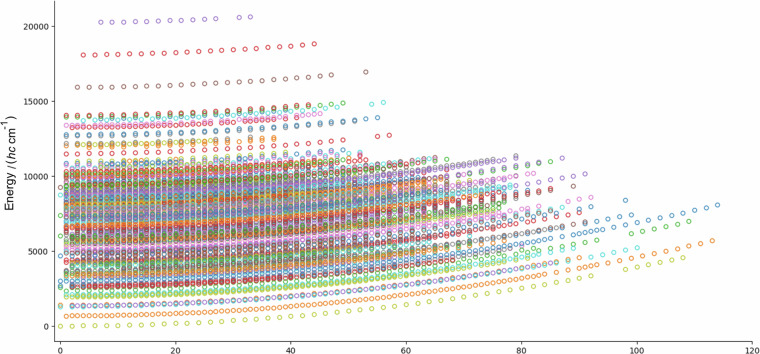
Pictorial representation of the empirical rovibrational energy levels of the ^16^O^12^C^16^O molecule determined in this study, as a function of the rotational quantum number *J* and the vibrational states (different colors refer to different vibrational states).

Figure 3 shows the empirical rovibrational energy levels of the ^16^O^12^C^16^O molecule determined in this study as a function of the rotational quantum number *J* and the total energy; the vibrational structure can also be seen in the quadratic curves formed as a function of *J*. Figure 3 shows that the list of rotational energy levels for the ground vibrational state extends up to *J* = 108, but is incomplete, as the *J* = 94 and 96 states are not present in the MARVEL energy levels.

Table 2 lists the vibrational band origins of ^16^O^12^C^16^O determined in this study; as *J* = 0 levels only exist for vibrational states with *ℓ*_2_ = 0, these band origins are only for vibrational states which have *ℓ*_2_ = 0. It is perhaps surprising to see that there are only nine energies listed there and only four of them have an accuracy better than 1 × 10^−3 ^*hc* cm^−1^.

**Table 2:** Empirical vibrational band origins of ^16^O^12^C^16^O determined in this study (part of the 626M24 dataset) and their counterparts in the CDSD-296^21^ and NASA Ames-2021^22^ databases.

Energy level	626M24 energy	CDSD-296 energy	Ames-2021 energy
(0 1 0 0 0 2 e)	1285.408 232(10)	1285.408 208	1285.414 232
(0 1 0 0 0 1 e)	1388.184 211(10)	1388.184 188	1388.186 563
(0 2 0 0 0 3 e)	2548.363 911(1515)	2548.366 693	2548.365 896
(0 2 0 0 0 2 e)	2671.143 110(1118)	2671.142 998	2671.139 583
(0 0 1 1 1 1 e)	3004.012 270(23)	—	—
(0 0 0 0 2 1 e)	4673.325 382(1)	4673.325 381	4673.299 366
(0 1 0 0 2 1 e)	6016.692 186(1000)	6016.690 087	6016.703 928
(0 2 0 0 2 1 e)	7377.705 281(1000)	—	—
(0 3 0 0 2 1 e)	8756.788 201(1000)	—	—
(0 0 0 0 4 1 e)	9246.928 103(5099)	—	—

All energy data are in *hc* cm^−1^.

## Data Availability

The developer version of the MARVEL code, used during the compilation and validation of the 626M24 dataset, is freely available as an OSF repository^184^.

## References

[1] Pearson, P. N. & Palmer, M. R. Atmospheric carbon dioxide concentrations over the past 60 million years. *Nature* **406**, 695–699, 10.1038/35021000 (2000). 10963587 10.1038/35021000

[2] Romps, D. M., Seeley, J. T. & Edman, J. P. Why the forcing from carbon dioxide scales as the logarithm of its concentration. *J. Climate* **35**, 4027–4047, 10.1175/JCLI-D-21-0275.1 (2022).

[3] Shine, K. P. & Perry, G. E. Radiative forcing due to carbon dioxide decomposed into its component vibrational bands. *Q. J. R. Meteorol.* **149**, 1856–1866, 10.1002/qj.4485 (2023).

[4] Calvin, K. *et al*. IPCC, 2023: Climate change 2023: Synthesis report. contribution of working groups I, II and III to the sixth assessment report of the intergovernmental panel on climate change [core writing team, H. Lee and J. Romero (eds.)]. IPCC, Geneva, Switzerland, Tech. rep. (2023).

[5] Baylis-Aguirre, D. K., Creech-Eakman, M. J. & Güth, T. Mid-IR spectra of the M-type Mira variable R Tri observed with the Spitzer IRS. *Mon. Not. Roy. Astron. Soc.* **493**, 807–814, 10.1093/mnras/staa322 (2020).

[6] Fonfria, J. P., Montiel, E. J., Cernicharo, J., DeWitt, C. N. & Richter, M. J. Detection of infrared fluorescence of carbon dioxide in R Leonis with SOFIA/EXES. *Astron. Astrophys.* **643**, L15, 10.1051/0004-6361/202039547 (2020). 33239827 10.1051/0004-6361/202039547 PMC7116412

[7] Gilli, G. *et al*. Limb observations of CO_2_ and CO non-LTE emissions in the Venus atmosphere by VIRTIS/Venus Express. *J. Geophys. Res.-Planets* **114**, E00B29, 10.1029/2008JE003112 (2009).

[8] Snels, M., Stefani, S., Grassi, D., Piccioni, G. & Adriani, A. Carbon dioxide opacity of the Venus’ atmosphere. *Planet Space Sci.* **103**, 347–354, 10.1016/j.pss.2014.08.002 (2014).

[9] Trokhimovskiy, A. *et al*. First observation of the magnetic dipole CO_2_ main isotopologue absorption band at 3.3 μm in the atmosphere of Mars by the ExoMars Trace Gas Orbiter ACS instrument. *Astron. Astrophys.* **639**, A142, 10.1051/0004-6361/202038134 (2020).

[10] Swain, M. R. *et al*. Molecular signatures in the near-infrared dayside spectrum of HD 189733b. *Astrophys. J. Lett.* **690**, L114–L117, 10.1088/0004-637X/690/2/L114 (2009).

[11] Ahrer, E.-M. *et al*. Identification of carbon dioxide in an exoplanet atmosphere. *Nature* **614**, 649–652, 10.1038/s41586-022-05269-w (2023). 36055338 10.1038/s41586-022-05269-w PMC9946830

[12] Boonman, A. M. S. *et al*. Gas-phase , , and HCN toward Orion-KL*. *Astron. Astrophys.* **399**, 1047–1061, 10.1051/0004-6361:20021799 (2003).

[13] Boonman, A. M. S., van Dishoeck, E. F., Lahuis, F. & Doty, S. D. Gas-phase CO_2_ toward massive protostars. *Astron. Astrophys.* **399**, 1063–1072, 10.1051/0004-6361:20021868 (2003).

[14] Hobbs, J. M. *et al*. Spectroscopic uncertainty impacts on OCO-2/3 retrievals of XCO_2_. *J. Quant. Spectrosc. Radiat. Transf.* **257**, 107360, 10.1016/j.jqsrt.2020.107360 (2020).

[15] Noel, S. *et al*. Greenhouse gas retrievals for the CO2M mission using the FOCAL method: first performance estimates. *Atmos. Meas. Tech.* **17**, 2317–2334, 10.5194/amt-17-2317-2024 (2024).

[16] Laughner, J. L. *et al*. The Total Carbon Column Observing Network’s GGG2020 data version. *Earth Sys. Sci. Data* **16**, 2197–2260, 10.5194/essd-16-2197-2024 (2024).

[17] Ageev, V. G. & Nikiforova, O. Y. Optoacoustic determination of carbon dioxide concentration in exhaled breath in various human diseases. *J. Appl. Spectrosc.* **83**, 820–825, 10.1007/s10812-016-0369-z (2016).

[18] Evseev, V., Fateev, A. & Clausen, S. High-resolution transmission measurements of CO_2_ at high temperatures for industrial applications. *J. Quant. Spectrosc. Radiat. Transf.* **113**, 2222–2233, 10.1016/j.jqsrt.2012.07.015 (2012).

[19] Du, Y., Tsankov, T. V., Luggenhöelscher, D. & Czarnetzki, U. Time evolution of CO_2_ ro-vibrational excitation in a nanosecond discharge measured with laser absorption spectroscopy. *J. Phys.D-Appl. Phys.* **54**, 365201, 10.1088/1361-6463/ac03e7 (2021).

[20] Snoeckx, R. & Bogaerts, A. Plasma technology - a novel solution for CO_2_ conversion? *Chem. Soc. Rev.* **46**, 5805–5863, 10.1039/c6cs00066e (2017). 28825736 10.1039/c6cs00066e

[21] Tashkun, S. A., Perevalov, V. I., Gamache, R. R. & Lamouroux, J. CDSD-296, high-resolution carbon dioxide spectroscopic databank: An update. *J. Quant. Spectrosc. Radiat. Transf.* **228**, 124–131, 10.1016/j.jqsrt.2019.03.001 (2019).

[22] Huang, X., Schwenke, D. W., Freedman, R. S. & Lee, T. J. Ames-2021 CO_2_ dipole moment surface and IR line lists: Toward 0.1% uncertainty for CO_2_ IR intensities. *J. Phys. Chem. A* **126**, 5940–5964, 10.1021/acs.jpca.2c01291 (2022). 36007245 10.1021/acs.jpca.2c01291

[23] Gordon, I. E. *et al*. The HITRAN2020 molecular spectroscopic database. *J. Quant. Spectrosc. Radiat. Transf.* **277**, 107949, 10.1016/j.jqsrt.2021.107949 (2022). 10.1016/j.jqsrt.2021.107735 PMC10408379 37554518

[24] Yurchenko, S. N., Mellor, T. M., Freedman, R. S. & Tennyson, J. ExoMol molecular line lists XXXIX: Ro-vibrational molecular line list for CO_2_. *Mon. Not. Roy. Astron. Soc.* **496**, 5282–5291, 10.1093/mnras/staa1874 (2020).

[25] Alatoom, D. *et al*. MARVEL analysis of high-resolution rovibrational spectra of ^16^O^12^C^18^O. *J. Comput. Chem.* **45**, 2558-2573, 10.1002/jcc.27453 (2024). 38997238 10.1002/jcc.27453

[26] Ibrahim, M. T. I. *et al*. MARVEL analysis of high-resolution rovibrational spectra of ^16^O^13^C^16^O. *J. Comput. Chem.* **45**, 969–984, 10.1002/jcc.27266 (2024). 38189163 10.1002/jcc.27266

[27] Azzam, A. A. A., Tennyson, J., Yurchenko, S. N., Furtenbacher, T. & Császár, A. G. MARVEL analysis of high-resolution rovibrational spectra of ^16^O^13^C^18^O, *J. Comput. Chem*. **46**, e27541, 10.1002/jcc.27541 (2025). 10.1002/jcc.27541 PMC11656679 39699052

[28] Azzam, A. A. A. *et al*. MARVEL analysis of high-resolution rovibrational spectra of the ^18^O^12^C^18^O, ^17^O^12^C^18^O, and ^18^O^13^C^18^O isotopologues of carbon dioxide. *J. Mol. Spectrosc.* **405**, 111947, 10.1016/j.jms.2024.111947 (2024).

[29] Császár, A. G., Czakó, G., Furtenbacher, T. & Mátyus, E. An active database approach to complete rotational–vibrational spectra of small molecules. *Annu. Rep. Comput. Chem.* **3**, 155–176, 10.1016/S1574-1400(07)03009-5 (2007).

[30] Furtenbacher, T., Császár, A. G. & Tennyson, J. MARVEL: measured active rotational-vibrational energy levels. *J. Mol. Spectrosc.* **245**, 115–125, 10.1016/j.jms.2007.07.005 (2007). 10.1038/s42004-026-02031-5 PMC13332032 42050102

[31] Furtenbacher, T. & Császár, A. G. MARVEL: measured active rotational-vibrational energy levels. II. Algorithmic improvements. *J. Quant. Spectrosc. Radiat. Transf.* **113**, 929–935, 10.1016/j.jqsrt.2012.01.005 (2012).

[32] Tennyson, J., Furtenbacher, T., Yurchenko, S. N. & Császár, A. G. Empirical rovibrational energy levels for nitrous oxide. *J. Quant. Spectrosc. Radiat. Transf.* **316**, 108902, 10.1016/j.jqsrt.2024.108902 (2024).

[33] Császár, A. G. & Furtenbacher, T. Spectroscopic networks. *J. Mol. Spectrosc.* **266**, 99 – 103, 10.1016/j.jms.2011.03.031 (2011).

[34] Furtenbacher, T. & Császár, A. G. The role of intensities in determining characteristics of spectroscopic networks. *J. Mol. Struct.* **1009**, 123–129, 10.1016/j.molstruc.2011.10.057 (2012).

[35] Tennyson, J. *et al*. The 2024 release of the ExoMol database: molecular line lists for exoplanet and other hot atmospheres. *J. Quant. Spectrosc. Radiat. Transf.* **326**, 109083, 10.1016/j.jqsrt.2024.109083 (2024).

[36] Martin, P. E. & Barker, E. F. The infrared absorption spectrum of carbon dioxide. *Phys. Rev.* **41**, 291–303, 10.1103/PhysRev.41.291 (1932).

[37] Nielsen, A. H. & Yao, Y. T. The analysis of the vibration-rotation band *ω*_3_ for and . *Phys. Rev.* **68**, 173–180, 10.1103/PhysRev.68.173 (1945).

[38] Goldberg, L., Mohler, O. C., Mcmath, R. R. & Pierce, A. K. Carbon dioxide in the infra-red solar spectrum. *Phys. Rev.* **76**, 1848–1858, 10.1103/PhysRev.76.1848 (1949).

[39] Herzberg, G. & Herzberg, L. Rotation-vibration spectra of diatomic and simple polyatomic molecules with long absorbing paths XI. The spectrum of carbon dioxide (CO_2_) below 1.25 *μ*m. *J. Opt. Soc. Am.* **43**, 1037–1044, 10.1364/JOSA.43.001037 (1953).

[40] Rossmann, K., Rao, K. N. & Nielsen, H. H. Infrared spectrum and molecular constants of carbon dioxide. Part I. *ν*_2_ of ^12^C^16^O_2_ at 15 μ. *J. Chem. Phys.* **24**, 103–105, 10.1063/1.1700807 (1956).

[41] Madden, R. P. A high-resolution study of CO_2_ absorption spectra between 15 and 18 microns. *J. Chem. Phys.* **35**, 2083–2097, 10.1063/1.1732212 (1961).

[42] Plyler, E. K., Tidwell, E. D. & Benedict, W. S. Absorption bands of carbon dioxide from 2.8–4.2 μ. *J. Opt. Soc. Am.* **52**, 1017–1022, 10.1364/JOSA.52.001017 (1962).

[43] Gordon, H. R. & McCubbin, T. K. The 15-micron bands of ^12^C^16^O_2_. *J. Mol. Spectrosc.* **18**, 73–82, 10.1016/0022-2852(65)90063-9 (1965).

[44] Gordon, H. R. The infrared spectrum of CO_2_ in the 2.8 and 15 micron regions, Ph.D. thesis, The Pennsylvania State University https://www.proquest.com/openview/59dcaa567b3cb6b908786d3699a70cc9/1?pq-origsite=gscholar&cbl=18750&diss=y (1965).

[45] Gordon, H. R. & McCubbin, T. K. The 2.8-micron bands of CO_2_. *J. Mol. Spectrosc.* **19**, 137–154, 10.1016/0022-2852(66)90237-2 (1966).

[46] Hartmann, B. & Kleman, B. Laser lines from CO_2_ in the 11-18 micron region. *Can. J. Phys.* **44**, 1609–1612, 10.1139/p66-134 (1966).

[47] Hahn, Y. H. The absorption and emission spectra of carbon-dioxide at 4.3 microns, Ph.D. thesis, The Pennsylvania State University https://www.proquest.com/openview/074bf2696a7b47d2793ca1d620a02193/1?pq-origsite=gscholar&cbl=18750&diss=y (1967).

[48] Oberly, R., Rao, K. N., Hahn, Y. H. & McCubbin, T. K. Bands of carbon dioxide in the region of 4.3 microns. *J. Mol. Spectrosc.* **25**, 138–165, 10.1016/0022-2852(68)80002-5 (1968).

[49] Gray Young, L. D., Young, A. T. & Schorn, R. A. Improved constants for the 7820 Å and 7883 Å bands of CO_2_. *J. Quant. Spectrosc. Radiat. Transfer* **10**, 1291–1300, 10.1016/0022-4073(70)90011-7 (1970).

[50] Blaney, T. G. *et al*. Absolute frequency measurement of the R(12) Transition of CO_2_ at 9.3 μm. *Nature* **244**, 504–504, 10.1038/244504a0 (1973).

[51] Evenson, K. M., Wells, J. S., Petersen, F. R., Danielson, B. L. & Day, G. W. Accurate frequencies of molecular transitions used in laser stabilization: the 3.39-μm transition in CH_4_ and the 9.33- and 10.18-*μ*m transitions in CO_2_. *Appl. Phys. Lett.* **22**, 192–195, 10.1063/1.1654607 (1973).

[52] Schiffner, G. Improved determination of accurate CO_2_ laser transition frequencies and their standard deviation. *Opto-Electronics* **5**, 411–413, 10.1007/bf01418076 (1973).

[53] McCubbin, T. K., Pliva, J., Pulfrey, R., Telfair, W. & Todd, T. The emission spectrum of ^12^C^16^O_2_ from 4.2 to 4.7 microns. *J. Mol. Spectrosc.* **49**, 136–156, 10.1016/0022-2852(74)90103-9 (1974).

[54] Toth, R. A. Wavenumbers, strengths, and self-broadened widths of CO_2_ at 3 μm. *J. Mol. Spectrosc.* **53**, 1–14, 10.1016/0022-2852(74)90256-2 (1974).

[55] Dupre-Maquaire, J. & Pinson, P. Emission spectrum of CO_2_ in the 9.6 μm region. *J. Mol. Spectrosc.* **62**, 181–191, 10.1016/0022-2852(76)90348-9 (1976).

[56] Reid, J. & Siemsen, K. New CO_2_ laser bands in the 9-11-μm wavelength region. *Appl. Phys. Lett.* **29**, 250–251, 10.1063/1.89033 (1976).

[57] Monchalin, J. P., Kelly, M. J., Thomas, J. E., Kurnit, N. A. & Javan, A. Accurate wavelength measurement of *P*-branch transitions of the band of ^12^C^16^O_2_ and determination of the band parameters. *J. Mol. Spectrosc.* **64**, 491–494, 10.1016/0022-2852(77)90233-8 (1977).

[58] Nereson, N. G. & Flicker, H. Wavenumber measurement of weak CO_2_ laser lines around 10.6 μm. *Opt. Commun.* **23**, 171–176, 10.1016/0030-4018(77)90299-1 (1977).

[59] Whitford, B. G., Siemsen, K. J. & Reid, J. Heterodyne frequency measurements of CO_2_ laser hot-band transitions. *Opt. Commun.* **22**, 261–264, 10.1016/S0030-4018(97)90004-3 (1977).

[60] Siemens, K. J. & Whitford, B. G. Heterodyne frequency measurements of CO_2_ laser sequence-band transitions. *Opt. Commun.* **22**, 11–16, 10.1016/0030-4018(77)90235-8 (1977).

[61] Arcas, P. & Arié, E. Absorption spectrum of CO_2_ in the 4.82-μm region. *J. Mol. Spectrosc.* **70**, 134–142, 10.1016/0022-2852(78)90015-2 (1978).

[62] Baldacci, A., Devi, V. M., Chen, D.-W., Rao, K. N. & Fridovich, B. Absorption spectrum of carbon dioxide at 4.3 μm. *J. Mol. Spectrosc.* **70**, 143–159, 10.1016/0022-2852(78)90016-4 (1978).

[63] Roney, P. L., Findlay, F. D., Buijs, H. L., Cann, M. W. P. & Nicholls, R. W. Carbon dioxide spectral line frequencies for the 43-μm region. *Appl. Opt.* **17**, 2599, 10.1364/AO.17.002599 (1978). 20203828 10.1364/AO.17.002599

[64] Freed, C., Bradley, L. & O’Donnell, R. Absolute frequencies of lasing transitions in seven CO_2_ isotopic species. *IEEE J. Quantum Electron.* **16**, 1195–1206, 10.1109/jqe.1980.1070392 (1980).

[65] Guelachvili, G. High-resolution Fourier spectra of carbon dioxide and three of its isotopic species near 4.3 μm. *J. Mol. Spectrosc.* **79**, 72–83, 10.1016/0022-2852(80)90293-3 (1980).

[66] Maillard, J. P., Cuisenier, M., Arcas, P., Arié, E. & Amiot, C. Infrared spectrum and molecular constants of CO_2_ in the 1.4-1.7 μm atmospheric window by very high resolution Fourier transform spectroscopy. *Can. J. Phys.* **58**, 1560–1569, 10.1139/p80-205 (1980).

[67] Paso, R., Kauppinen, J. & Anttila, R. Infrared spectrum of CO_2_ in the region of the bending fundamental *ν*_2_. *J. Mol. Spectrosc.* **79**, 236–253, 10.1016/0022-2852(80)90304-5 (1980).

[68] Pine, A. S. & Guelachvili, G. R-branch head of the *ν*_3_ band of CO_2_ at elevated temperatures. *J. Mol. Spectrosc.* **79**, 84–89, 10.1016/0022-2852(80)90294-5 (1980).

[69] Bailly, D., Farrenq, R., Guelachvili, G. & Rossetti, C. ^12^C^16^O_2_ analysis of emission Fourier spectra in the 4.5-*μ*m region: Rovibrational transitions . *J. Mol. Spectrosc.* **90**, 74–105, 10.1016/0022-2852(81)90334-9 (1981).

[70] Kauppinen, J., Jolma, K. & Horneman, V. M. New wave-number calibration tables for H_2_O, CO_2_, and OCS lines between 500 and 900 cm^−1^. *Appl Opt.* **21**, 3332–6, 10.1364/AO.21.003332 (1982). 20396232 10.1364/AO.21.003332

[71] Arcas, P., Arie, E., Cuisenier, M. & Maillard, J. P. The infrared spectrum and molecular constants of CO_2_ in the 2 μm region. *Can. J. Phys.* **61**, 857–866, 10.1139/p83-105 (1983).

[72] Jolma, K., Kauppinen, J. & Horneman, V. M. Vibration-rotation bands of CO_2_ and OCS in the region 540-890 cm^−1^. *J. Mol. Spectrosc.* **101**, 300–305, 10.1016/0022-2852(83)90135-2 (1983).

[73] Petersen, F. R., Beaty, E. C. & Pollock, C. R. Improved rovibrational constants and frequency tables for the normal laser bands of ^12^C^16^O_2_. *J. Mol. Spectrosc.* **102**, 112–122, 10.1016/0022-2852(83)90231-X (1983).

[74] Devi, V. M., Rinsland, C. P. & Benner, D. C. Absolute intensity measurements of CO_2_ bands in the 2395-2680-cm^−1^ region. *Appl. Opt.* **23**, 4067, 10.1364/AO.23.004067 (1984). 18213277 10.1364/ao.23.004067

[75] Rinsland, C. P. & Benner, D. C. Absolute intensities of spectral lines in carbon dioxide bands near 2050 cm^−1^. *Appl. Opt.* **23**, 4523–4528, 10.1364/AO.23.004523 (1984). 18213344 10.1364/ao.23.004523

[76] Rinsland, C. P. *et al*. Atlas of high resolution infrared spectra of carbon dioxide. *Appl. Opt.* **23**, 2051–2052, 10.1364/AO.23.002051 (1984). 20424724 10.1364/AO.23.002051

[77] Petersen, F. R., Wells, J. S., Siemsen, K. J., Robinson, A. M. & Maki, A. G. Heterodyne frequency measurements and analysis of CO_2_ laser hot band transitions. *J. Mol. Spectrosc.* **105**, 324–330, 10.1016/0022-2852(84)90222-4 (1984).

[78] Benner, D. C. & Rinsland, C. P. Identification and intensities of the “forbidden” 3 band of ^12^C^16^O_2_. *J. Mol. Spectrosc.* **112**, 18–25, 10.1016/0022-2852(85)90187-0 (1985).

[79] Brown, L. R. & Toth, R. A. Comparison of the frequencies of NH_3_, CO_2_, H_2_O, N_2_O, CO, and CH_4_ as infrared calibration standards. *J. Opt. Soc. Am. B* **2**, 842–856, 10.1364/JOSAB.2.000842 (1985).

[80] Rinsland, C. P., Benner, D. C. & Devi, V. M. Measurements of absolute line intensities in carbon dioxide bands near 5.2 μm. *Appl. Opt.* **24**, 1644–1650, 10.1364/AO.24.001644 (1985). 18223769 10.1364/ao.24.001644

[81] Bradley, L., Soohoo, K. & Freed, C. Absolute frequencies of lasing transitions in nine CO_2_ isotopic species. *IEEE J. Quantum Electron.* **22**, 234–267, 10.1109/JQE.1986.1072967 (1986).

[82] Chardonnet, C., van Lerberghe, A. & Bordé, C. J. Absolute frequency determination of super-narrow CO_2_ saturation peaks observed in an external absorption cell. *Opt. Commun.* **58**, 333–337, 10.1016/0030-4018(86)90239-7 (1986).

[83] Esplin, M. P. & Rothman, L. S. Spectral measurements of high-temperature isotopic carbon dioxide in the 4.5- and 2.8 μm regions. *J. Mol. Spectrosc.* **116**, 351–363, 10.1016/0022-2852(86)90132-3 (1986).

[84] Esplin, M. P. *et al*. Carbon dioxide line positions in the 2.8 and 4.3 micron regions at 800 Kelvin, Tech. Rep. AFGL-TR-86-0046, Utah State University https://apps.dtic.mil/sti/citations/ADA173808 (1986).

[85] Guelachvili, G., Rao, K. R. Handbook of Infrared Standards, Academic Press, (1986).

[86] Rinsland, C. P., Benner, D. C. & Devi, V. M. Absolute line intensities in CO_2_ bands near 4.8 μm. *Appl. Opt.* **25**, 1204–1214, 10.1364/AO.25.001204 (1986). 18231320 10.1364/ao.25.001204

[87] Blanquet, G., Walrand, J. & Teffo, J. L. Frequency diode laser measurement of a very weak Q branch of CO_2_ near 864 cm^−1^. *Appl. Opt.* **27**, 2098–2099, 10.1364/ao.27.002098 (1988). 20531716 10.1364/AO.27.002098

[88] Benner, D. C., Devi, V. M., Rinsland, C. P. & Ferry-Leeper, P. S. Absolute intensities of CO_2_ lines in the 3140-3410-cm^−1^ spectral region. *Appl. Opt.* **27**, 1588–1597, 10.1364/AO.27.001588 (1988). 20531618 10.1364/AO.27.001588

[89] Ouazzany, Y., Boquillon, J. P. & Schrötter, H. W. High resolution CARS spectrum and analysis of the *ν*_1_ band Q-branch of carbon dioxide. *Mol. Phys.* **63**, 769–777, 10.1080/00268978800100551 (1988).

[90] Hamdouni, A. & Dana, V. Absolute line intensities in the 20002 ← 11102 and 12201 ← 03301 bands of ^12^C^16^O_2_. *Appl. Opt.* **29**, 1570–1572, 10.1364/AO.29.001570 (1990). 20563041 10.1364/AO.29.001570

[91] Giver, L. P. & Chackerian, C. Rovibrational intensities for the band of ^12^C^16^O_2_ at 4416 cm^−1^. *J. Mol. Spectrosc.* **148**, 80–85, 10.1016/0022-2852(91)90036-a (1991).

[92] Groh, A., Goddon, D., Schneider, M., Zimmermann, W. & Urban, W. Sub-doppler heterodyne frequency measurements on the CO_2_ 10011-00001 vibrational band: New reference lines near 3714 cm^−1^. *J. Mol. Spectrosc.* **146**, 161–168, 10.1016/0022-2852(91)90379-O (1991).

[93] Bailly, D. ^12^C^16^O_2_ in emission in the 4.5-*μ*m region: Some more rovibrational transitions occurring between very few populated levels. *J. Mol. Spectrosc.* **166**, 1–11, 10.1006/jmsp.1994.1166 (1994).

[94] Maki, A. G., Chou, C. C., Evenson, K. M., Zink, L. R. & Shy, J. T. Improved molecular constants and frequencies for the CO_2_ laser from new high-*J* regular and hot-band frequency measurements. *J. Mol. Spectrosc.* **167**, 211–224, 10.1006/jmsp.1994.1227 (1994).

[95] Chou, C. C. *et al*. Frequency measurements and molecular constants of CO_2_ 00^0^2-[10^0^1,02^0^1]_I,II_ sequence band transitions. *J. Mol. Spectrosc.* **172**, 233–242, 10.1006/jmsp.1995.1171 (1995).

[96] Steyert, D. W., Weber, M., Sirota, J. M. & Reuter, D. C. Absolute intensities for the Q-branch of the (581.776 cm^−1^) band in carbon dioxide. *J. Quant. Spectrosc. Rad. Transf.* **54**, 815–818, 10.1016/0022-4073(95)00101-p (1995).

[97] Bailly, D., Camy-Peyret, C. & Lanquetin, R. Temperature measurement in flames through CO_2_ and CO emission: New highly excited levels of CO_2_. *J. Mol. Spectrosc.* **182**, 10–17, 10.1006/jmsp.1996.7205 (1997).

[98] Bernard, V., Nogues, G., Daussy, C., Constantin, L. & Chardonnet, C. CO_2_ laser stabilized on narrow saturated absorption resonances of CO_2_, improved absolute frequency measurements. *Metrologia* **34**, 313, 10.1088/0026-1394/34/4/4 (1997).

[99] Bailly, D. ^12^C^16^O_2_ in emission in the 4.5-*μ*m region: Transitions with (2*v*_1_ + *v*_2_) = 6 occurring between highly excited vibrational states. *J. Mol. Spectrosc.* **192**, 257–262, 10.1006/jmsp.1998.7667 (1998). 10.1006/jmsp.1998.7667 9831492

[100] Bailly, D., Tashkun, S. A., Perevalov, V. I., Teffo, J. L. & Arcas, P. H. CO_2_ emission in the 4-μm region: The transition revisited. *J. Mol. Spectrosc.* **190**, 1–6, 10.1006/jmsp.1998.7534 (1998). 10.1006/jmsp.1998.7534 9645923

[101] Devi, V. M., Benner, D. C., Rinsland, C. P. & Smith, M. A. H. Absolute rovibrational intensities of ^12^C^16^O_2_ absorption bands in the 3090-3850 cm^−1^ spectral region. *J. Quant. Spectrosc. Rad. Transf.* **60**, 741–770, 10.1016/s0022-4073(98)00080-6 (1998).

[102] Frech, B. *et al*. Frequency measurements of saturated-fluorescence-stabilized CO_2_ laser lines: comparison with an OsO_4_-stabilized CO_2_ laser standard. *Appl. Phys. B* **67**, 217–221, 10.1007/s003400050496 (1998).

[103] Bailly, D., Tashkun, S., Perevalov, V. I., Teffo, J. L. & Arcas, P. Flame spectra of CO_2_ in the 3-μm region. *J. Mol. Spectrosc.* **197**, 114–119, 10.1006/jmsp.1999.7883 (1999). 10438647 10.1006/jmsp.1999.7883

[104] Campargue, A., Bailly, D., Teffo, J. L., Tashkun, S. A. & Perevalov, V. I. The *ν*_1_ + 5*ν*_3_ dyad of ^12^CO_2_ and ^13^CO_2_. *J. Mol. Spectrosc.* **193**, 204–212, 10.1006/jmsp.1998.7718 (1999). 9878501 10.1006/jmsp.1998.7718

[105] Acef, O., Michaud, F. & Rovera, G. V. Accurate determination of OsO_4_ absolute frequency grid at 28/29 THz. *IEEE Trans. Instr. Meas.* **48**, 567–570 (1999).

[106] Predoi-Cross, A., Luo, C., Berman, R., Drummond, J. R. & May, A. D. Line strengths, self-broadening, and line mixing in the 200 ← 01^1^0 (*Σ* ← *Π*) Q branch of carbon dioxide. *J. Chem. Phys.* **112**, 8367–8377, 10.1063/1.481480 (2000).

[107] Tashkun, S. A. *et al*. ^13^C^16^O_2_: Global treatment of vibrational-rotational spectra and first observation of the 2*ν*_1_ + 5*ν*_3_ and *ν*_1_ + 2*ν*_2_ + 5*ν*_3_ absorption bands. *J. Mol. Spectrosc.* **200**, 162–176, 10.1006/jmsp.2000.8057 (2000). 10708529 10.1006/jmsp.2000.8057

[108] Teffo, J.-L. *et al*. Line intensities of ^12^C^16^O_2_ in the 1.2-1.4 μm spectral region. *J. Mol. Spectrosc.* **201**, 249–255, 10.1006/jmsp.2000.8092 (2000). 10814487 10.1006/jmsp.2000.8092

[109] Chou, C.-C., Lin, T. & Shy, J.-T. Wavenumber measurements of CO_2_ transitions in 1.5-μm atmospheric window using an external-cavity diode laser. *J. Mol. Spectrosc.* **205**, 122–127, 10.1006/jmsp.2000.8242 (2001). 11148116 10.1006/jmsp.2000.8242

[110] Weirauch, G. & Campargue, A. Spectroscopy and intensity measurements of the 3*ν*_1_+3*ν*_3_ tetrad of ^12^CO_2_ and ^13^CO_2_. *J. Mol. Spectrosc.* **207**, 263–268, 10.1006/jmsp.2001.8342 (2001). 11397115 10.1006/jmsp.2001.8342

[111] Vander Auwera, J., El Hachtouki, R. & Brown, L. R. Absolute line wavenumbers in the near infrared: ^12^C_2_H_2_ and ^12^C^16^O_2_. *Mol. Phys.* **100**, 3563–3576, 10.1080/00268970210162880 (2002).

[112] Ding, Y., Bertseva, E. & Campargue, A. Note: The 2*ν*_1_ + 3*ν*_3_ triad of ^12^CO_2_. *J. Mol. Spectrosc.* **212**, 219–222, 10.1006/jmsp.2002.8553 (2002).

[113] Devi, V. M., Benner, D. C., Smith, M. A. H. & Rinsland, C. P. Nitrogen broadening and shift coeffcients in the 4.2-4.5 micron bands of CO_2_. *J. Quant. Spectrosc. Rad. Transf.* **76**, 289–307 (2003).

[114] Giver, L. P., Brown, L. R., Chackerian, C. & Freedman, R. S. The rovibrational intensities of five absorption bands of between 5218 and 5349 cm^−1^. *J. Quant. Spectrosc. Rad. Transf.* **78**, 417–436, 10.1016/s0022-4073(02)00277-7 (2003).

[115] Miller, C. E. & Brown, L. R. Near infrared spectroscopy of carbon dioxide I. ^16^O^12^C^16^O line positions. *J. Mol. Spectrosc.* **228**, 329–354, 10.1016/j.jms.2003.11.001 (2004).

[116] André, F., Perrin, M. Y. & Taine, J. FTIR measurements of ^12^C^16^O_2_ line positions and intensities at high temperature in the 3700-3750 cm^−1^ spectral region. *J. Mol. Spectrosc.* **228**, 187–205, 10.1016/j.jms.2004.07.004 (2004).

[117] Amy-Klein, A., Vigué, H. & Chardonnet, C. Absolute frequency measurement of ^12^C^16^O_2_ laser lines with a femtosecond laser comb and new determination of the ^12^C^16^O_2_ molecular constants and frequency grid. *J. Mol. Spectrosc.* **228**, 206–212, 10.1016/j.jms.2004.07.005 (2004).

[118] Ding, Y., Campargue, A., Bertseva, E., Tashkun, S. & Perevalov, V. I. Highly sensitive absorption spectroscopy of carbon dioxide by ICLAS-VeCSEL between 8800 and 9530 cm^−1^. *J. Mol. Spectrosc.* **231**, 117–123, 10.1016/j.jms.2004.12.008 (2005).

[119] Garnache, A., Liu, A., Cerutti, L. & Campargue, A. Intracavity laser absorption spectroscopy with a vertical external cavity surface emitting laser at 2.3 μm: Application to water and carbon dioxide. *Chem. Phys. Lett.* **416**, 22–27, 10.1016/j.cplett.2005.09.028 (2005).

[120] Lucchesini, A. & Gozzini, S. Diode laser overtone spectroscopy of CO_2_ at 780 nm. *J. Quant. Spectrosc. Radiat. Transf.* **96**, 289–299, 10.1016/j.jqsrt.2005.03.005 (2005).

[121] Mazzotti, D., Cancio, P., Giusfredi, G., De Natale, P. & Prevedelli, M. Frequency-comb-based absolute frequency measurements in the mid-infrared with a difference-frequency spectrometer. *Opt. Lett.* **30**, 997, 10.1364/ol.30.000997 (2005). 15906982 10.1364/ol.30.000997

[122] Majcherova, Z. *et al*. High-sensitivity CW-cavity ringdown spectroscopy of ^12^CO_2_ near 1.5 μm. *J. Mol. Spectrosc.* **230**, 1–21, 10.1016/j.jms.2004.09.011 (2005).

[123] Shao, J. *et al*. Highly sensitive diode laser absorption measurements of CO_2_ near 1.57 μm at room temperature. *Opt. Appl.* **XXXV**, 49–57 (2005).

[124] Wang, L., Perevalov, V. I., Tashkun, S. A., Liu, A. W. & Hu, S. M. Absorption spectra of ^12^C^16^O_2_ and ^13^C^16^O_2_ near 1.05 μm. *J. Mol. Spectrosc.* **233**, 297–300, 10.1016/j.jms.2005.07.008 (2005).

[125] Perevalov, B. V. *et al*. CW-cavity ringdown spectroscopy of carbon dioxide isotopologues near 1.5 μm. *J. Mol. Spectrosc.* **238**, 241–255, 10.1016/j.jms.2006.05.009 (2006).

[126] Toth, R. A., Brown, L. R., Miller, C. E., Devi, V. M. & Benner, D. C. Line strengths of ^12^C^16^O_2_ : 4550-7000 cm^−1^. *J. Mol. Spectrosc.* **239**, 221–242, 10.1016/j.jms.2006.08.001 (2006).

[127] Horneman, V.-M. High accurate peak positions for calibration purposes with the lowest fundamental bands *ν*_2_ of N_2_O and CO_2_. *J. Mol. Spectrosc.* **241**, 45–50, 10.1016/j.jms.2006.10.014 (2007).

[128] Lucchesini, A. & Gozzini, S. Diode laser spectroscopy of CO_2_ at 790 nm. *J. Quant. Spectrosc. Rad. Transf.* **103**, 74–82, 10.1016/j.jqsrt.2006.06.009 (2007).

[129] Borri, S. *et al*. Lamb-dip-locked quantum cascade laser for comb-referenced IR absolute frequency measurements. *Opt. Expr.* **16**, 11637, 10.1364/oe.16.011637 (2008). 10.1364/oe.16.011637 18648485

[130] Perevalov, B. V., Kassi, S., Perevalov, V. I., Tashkun, S. A. & Campargue, A. High sensitivity CW-CRDS spectroscopy of ^12^C^16^O_2_, ^16^O^12^C^17^O and ^16^O^12^C^18^O between 5851 and 7045 cm^−1^: Line positions analysis and critical review of the current databases. *J. Mol. Spectrosc.* **252**, 143–159, 10.1016/j.jms.2008.06.012 (2008).

[131] Kassi, S., Song, K. F. & Campargue, A. High sensitivity CW-cavity ring down spectroscopy of ^12^CO_2_ near 1.35 μm (I): line positions. *J. Quant. Spectrosc. Radiat. Transf.* **110**, 1801–1814, 10.1016/j.jqsrt.2009.04.010 (2009).

[132] Campargue, A., Song, K. F., Mouton, N., Perevalov, V. I. & Kassi, S. High sensitivity CW-Cavity Ring Down Spectroscopy of five ^13^CO_2_ isotopologues of carbon dioxide in the 1.26-1.44 *μ*m region (I): Line positions. *J. Quant. Spectrosc. Radiat. Transf.* **111**, 659–674, 10.1016/j.jqsrt.2009.11.013 (2010).

[133] Pastor, P. C., Galli, I., Giusfredi, G., Mazzotti, D., De Natale, P. Saturated-absorption cavity ring-down spectroscopy, in: Frontiers in Optics 2010/Laser Science XXVI, Vol. 104 of FiO, OSA, 2010, p. FTuL4. 10.1364/fio.2010.ftul4 10.1103/PhysRevLett.104.110801 20366460

[134] Song, K. F., Kassi, S., Tashkun, S. A., Perevalov, V. I. & Campargue, A. High sensitivity CW-cavity ring down spectroscopy of ^12^CO_2_ near 1.35 μm (II): New observations and line intensities modeling. *J. Quant. Spectrosc. Radiat. Transf.* **111**, 332–344, 10.1016/j.jqsrt.2009.09.004 (2010).

[135] Gatti, D. *et al*. Absolute frequency spectroscopy of CO_2_ lines at around 2.09 μm by combined use of an Er:fiber comb and a Ho:YLF amplifier. *Opt. Lett.* **36**, 3921–3, 10.1364/OL.36.003921 (2011). 21964142 10.1364/OL.36.003921

[136] Gatti, D. *et al*. High-precision molecular interrogation by direct referencing of a quantum-cascade-laser to a near-infrared frequency comb. *Opt. Expr.* **19**, 17520, 10.1364/oe.19.017520 (2011). 10.1364/OE.19.017520 21935118

[137] Song, K.-F. *et al*. High sensitivity cavity ring down spectroscopy of CO_2_ overtone bands near 790 nm. *J. Quant. Spectrosc. Rad. Transf.* **112**, 761–768, 10.1016/j.jqsrt.2010.11.006 (2011).

[138] Cappelli, F. *et al*. Subkilohertz linewidth room-temperature mid-infrared quantum cascade laser using a molecular sub-doppler reference. *Opt. Lett.* **37**, 4811, 10.1364/ol.37.004811 (2012). 23202054 10.1364/OL.37.004811

[139] Gambetta, A. *et al*. Comb-assisted spectroscopy of CO_2_ absorption profiles in the near- and mid-infrared regions. *Appl. Phys. B* **109**, 385–390, 10.1007/s00340-012-4947-3 (2012).

[140] Jacquemart, D. *et al*. Infrared spectroscopy of CO_2_ isotopologues from 2200 to 7000 cm^−1^: I—Characterizing experimental uncertainties of positions and intensities. *J. Quant. Spectrosc. Radiat. Transf.* **113**, 961–975, 10.1016/j.jqsrt.2012.02.020 (2012).

[141] Lyulin, O. M. *et al*. Infrared spectroscopy of ^17^O- and ^18^O-enriched carbon dioxide in the 1700-8300 cm^−1^ wavenumber region. *J. Quant. Spectrosc. Radiat. Transf.* **113**, 2167–2181, 10.1016/j.jqsrt.2012.06.028 (2012).

[142] Galli, I. *et al*. Absolute frequency measurements of CO_2_ transitions at 4.3 μm with a comb-referenced quantum cascade laser. *Mol. Phys.* **111**, 2041–2045, 10.1080/00268976.2013.782436 (2013).

[143] Liao, C.-C., Lien, Y.-H., Wu, K.-Y., Lin, Y.-R. & Shy, J.-T. Widely tunable difference frequency generation source for high-precision mid-infrared spectroscopy. *Opt. Expr.* **21**, 9238, 10.1364/oe.21.009238 (2013). 10.1364/OE.21.009238 23609634

[144] Lu, Y. *et al*. Line paremeters of the 782 nm band of CO_2_. *Astrophys. J.* **775**, 71, 10.1088/0004-637X/775/1/71 (2013).

[145] Petrova, T. M. *et al*. Measurements of ^12^C^16^O_2_ line parameters in the 8790-8860, 9340-9650 and 11,430-11,505 cm^−1^ wavenumber regions by means of Fourier transform spectroscopy. *J. Quant. Spectrosc. Rad. Transf.* **124**, 21–27, 10.1016/j.jqsrt.2013.03.017 (2013).

[146] Long, D. A., Truong, G.-W., Hodges, J. T. & Miller, C. E. Absolute ^12^C^16^O_2_ transition frequencies at the kHz-level from 1.6 to 7.8 μm. *J. Quant. Spectrosc. Rad. Transf.* **130**, 112–115, 10.1016/j.jqsrt.2013.07.001 (2013).

[147] Truong, G.-W. *et al*. Comb-linked, cavity ring-down spectroscopy for measurements of molecular transition frequencies at the kHz-level, *J. Chem. Phys*. 138. 10.1063/1.4792372 (2013) 10.1063/1.4792372 23485285

[148] Borkov, Y. G., Jacquemart, D., Lyulin, O. M., Tashkun, S. A. & Perevalov, V. I. Infrared spectroscopy of ^17^O- and ^18^O-enriched carbon dioxide: Line positions and intensities in the 3200–4700 cm^−1^ region. Global modeling of the line positions of ^16^O^12^C^17^O and ^17^O^12^C^17^O. *J. Quant. Spectrosc. Radiat. Transf.* **137**, 57–76, 10.1016/j.jqsrt.2013.11.008 (2014).

[149] Karlovets, E. V., Kassi, S., Tashkun, S. A., Perevalov, V. I. & Campargue, A. High sensitivity cavity ring down spectroscopy of carbon dioxide in the 1.19-1.26 μm region. *J. Quant. Spectrosc. Radiat. Transf.* **144**, 137–153, 10.1016/j.jqsrt.2014.04.001 (2014).

[150] Burkart, J. *et al*. Communication: Saturated CO_2_ absorption near 1.6 μm for kilohertz-accuracy transition frequencies. *J. Chem. Phys.* **142**, 191103, 10.1063/1.4921557 (2015). 26001440 10.1063/1.4921557

[151] Borkov, Y. G., Jacquemart, D., Lyulin, O. M., Tashkun, S. A. & Perevalov, V. I. Infrared spectroscopy of ^17^O- and ^18^O-enriched carbon dioxide: Line positions and intensities in the 4681-5337 cm^−1^ region. *J. Quant. Spectrosc. Radiat. Transf.* **159**, 1–10, 10.1016/j.jqsrt.2015.02.019 (2015).

[152] Gatti, D. *et al*. Comb-locked cavity ring-down spectrometer, *J. Chem. Phys*. 142 10.1063/1.4907939 (2015) 10.1063/1.4907939 25702008

[153] Lamouroux, J. *et al*. CO_2_ line-mixing database and software update and its tests in the 2.1 μm and 4.3 μm regions. *J. Quant. Spectrosc. Radiat. Transf.* **151**, 88–96, 10.1016/j.jqsrt.2014.09.017 (2015).

[154] Jacquemart, D., Borkov, Y. G., Lyulin, O. M., Tashkun, S. A. & Perevalov, V. I. Fourier transform spectroscopy of CO_2_ isotopologues at 1.6 μm: Line positions and intensities. *J. Quant. Spectrosc. Radiat. Transf.* **160**, 1–9, 10.1016/j.jqsrt.2015.03.016 (2015).

[155] Long, D. A., Wójtewicz, S., Miller, C. E. & Hodges, J. T. Frequency-agile, rapid scanning cavity ring-down spectroscopy (FARS-CRDS) measurements of the (30012) ← (00001) near-infrared carbon dioxide band. *J. Quant. Spectrosc. Rad. Transf.* **161**, 35–40, 10.1016/j.jqsrt.2015.03.031 (2015).

[156] Petrova, T. M. *et al*. Measurements of CO_2_ line parameters in the 9250–9500 cm^−1^ and 10,700–10,860 cm^−1^ regions. *J. Quant. Spectrosc. Radiat. Transf.* **164**, 109–116, 10.1016/j.jqsrt.2015.06.001 (2015).

[157] Tan, Y. *et al*. Cavity ring-down spectroscopy of CO_2_ overtone bands near 830 nm. *J. Quant. Spectrosc. Rad. Transf.* **165**, 22–27, 10.1016/j.jqsrt.2015.06.010 (2015).

[158] Benner, D. C. *et al*. Line parameters including temperature dependences of air- and self-broadened line shapes of ^12^C^16^O_2_: 2.06-μm region. *J. Mol. Spectrosc.* **326**, 21–47, 10.1016/j.jms.2016.02.012 (2016).

[159] Devi, V. M. *et al*. Line parameters including temperature dependences of self- and air-broadened line shapes of ^12^C^16^O_2_: 1.6-μm region. *J. Quant. Spectrosc. Radiat. Transf.* **177**, 117–144, 10.1016/j.jqsrt.2015.12.020 (2016).

[160] Vasilchenko, S. *et al*. The CO_2_ absorption spectrum in the 2.3 *μ*m transparency window by high sensitivity CRDS: (I) Rovibrational lines. *J. Quant. Spectrosc. Radiat. Transf.* **184**, 233–240, 10.1016/j.jqsrt.2016.07.002 (2016).

[161] Guan, Y.-C. *et al*. Frequency measurements and molecular constants of the ^12^C band near 2.7 μm. *J. Mol. Spectrosc.* **334**, 26–30, 10.1016/j.jms.2017.03.011 (2017).

[162] Kassi, S., Karlovets, E. V., Tashkun, S. A., Perevalov, V. I. & Campargue, A. Analysis and theoretical modeling of the ^18^O enriched carbon dioxide spectrum by CRDS near 1.35 μm: (I) ^16^O^12^C^18^O, ^16^O^12^C^17^O, ^12^C^16^O_2_ and ^13^C^16^O_2_. *J. Quant. Spectrosc. Radiat. Transf.* **187**, 414–425, 10.1016/j.jqsrt.2016.09.002 (2017).

[163] Čermák, P. *et al*. High sensitivity CRDS of CO_2_ in the 1.74 μm transparency window. A validation test for the spectroscopic databases. *J. Quant. Spectrosc. Radiat. Transf.* **207**, 95–103, 10.1016/j.jqsrt.2017.12.018 (2018).

[164] Karlovets, E. V. *et al*. Analysis and theoretical modeling of the ^18^O enriched carbon dioxide spectrum by CRDS near 1.74 μm. *J. Quant. Spectrosc. Radiat. Transf.* **217**, 73–85, 10.1016/j.jqsrt.2018.05.017 (2018).

[165] Gotti, R. *et al*. Comb-locked frequency-swept synthesizer for high precision broadband spectroscopy. *Sci. Rep.* **10**, 2523, 10.1038/s41598-020-59398-1 (2020). 32054902 10.1038/s41598-020-59398-1 PMC7018949

[166] Karlovets, E. V., Kassi, S. & Campargue, A. High sensitivity CRDS of CO_2_ in the 1.18 μm transparency window. Validation tests of current spectroscopic databases. *J. Quant. Spectrosc. Radiat. Transf.* **247**, 106942, 10.1016/j.jqsrt.2020.106942 (2020).

[167] Lamperti, M. *et al*. Optical frequency metrology in the bending modes region, *Commun. Phys*. 3 10.1038/s42005-020-00441-y (2020).

[168] Reed, Z. D., Long, D. A., Fleurbaey, H. & Hodges, J. T. SI-traceable molecular transition frequency measurements at the 10^−12^ relative uncertain level. *Optica* **7**, 1209–1220, 10.1364/OPTICA.395943 (2020).

[169] Wu, H. *et al*. A well-isolated vibrational state of CO_2_ verified by near-infrared saturated spectroscopy with kHz accuracy. *Phys. Chem. Chem. Phys.* **22**, 2841–2848, 10.1039/C9CP05121J (2020). 31967121 10.1039/c9cp05121j

[170] Predoi-Cross, A. & Buldyreva, J. Characterization of line mixing effects in the 11101 ← 00001 band of carbon dioxide for pressures up to 19 atm. *J. Quant. Spectrosc. Radiat. Transf.* **272**, 107793, 10.1016/j.jqsrt.2021.107793 (2021).

[171] Reed, Z. D., Drouin, B. J., Long, D. A. & Hodges, J. T. Molecular transition frequencies of CO_2_ near 1.6 μm with kHz-level uncertainties. *J. Quant. Spectrosc. Rad. Transf.* **271**, 107681, 10.1016/j.jqsrt.2021.107681 (2021).

[172] Marinina, A. A. *et al*. Absorption spectrum of carbon dioxide in the 4350-4550 cm^−1^ region. *Atmos. Ocean. Opt.* **35**, 8–13, 10.1134/s1024856022010109 (2022).

[173] Tan, Y. *et al*. Cavity-enhanced saturated absorption spectroscopy of the (30012) - (00001) band of ^12^C^16^O_2_. *J. Chem. Phys.* **156**, 044201, 10.1063/5.0074713 (2022). 35105067 10.1063/5.0074713

[174] Chen, T. Y., Steinmetz, S. A., Patterson, B. D., Jasper, A. W. & Kliewer, C. J. Direct observation of coherence transfer and rotational-to-vibrational energy exchange in optically centrifuged CO_2_ super-rotors. *Nat. Commun.* **14**, 3227, 10.1038/s41467-023-38873-z (2023). 37270647 10.1038/s41467-023-38873-z PMC10239519

[175] Fleurbaey, H. *et al*. ^12^CO_2_ transition frequencies with kHz-accuracy by saturation spectroscopy in the 1.99-2.09 μm region. *Phys. Chem. Chem. Phys.* **25**, 16319–16330, 10.1039/D3CP01603J (2023). 37309841 10.1039/d3cp01603j

[176] Lyulin, O. M., Solodov, A. M., Solodov, A. A., Petrova, T. M. & Perevalov, V. I. The absorption bands of ^12^C^16^O_2_ near 718 nm. *J. Quant. Spectrosc. Rad. Transf.* **303**, 108595, 10.1016/j.jqsrt.2023.108595 (2023).

[177] Birk, M., Röske, C. & Wagner, G. The pressure dependence of the experimentally-determined line intensity and continuum absorption of pure CO_2_ in the 1.6 μm region. *J. Quant. Spectrosc. Rad. Transf.* **324**, 109055, 10.1016/j.jqsrt.2024.109055 (2024).

[178] Jiang, S., Tan, Y., Liu, A.-W., Zhou, X.-G. & Hu, S.-M. Saturated cavity ring-down spectroscopy of ^12^C^16^O_2_ near 1.57 μm. *Chin. J. Chem. Phys.* **37**, 13–18, 10.1063/1674-0068/cjcp2305046 (2024).

[179] Ritter, M. E., DeSouza, S. A., Ogden, H. M., Michael, T. J. & Mullin, A. S. Transient IR spectroscopy of optically centrifuged CO_2_ (R186–R282) and collision dynamics for the *J* = 244 − 282 states. *Faraday Discuss.* **251**, 140–159, 10.1039/D3FD00179B (2024). 38766993 10.1039/d3fd00179b

[180] Serdyukov, V. I., Sinitsa, L. N. & Vasil’chenko, S. S. Highly sensitive Fourier transform spectroscopy with LED sources. *J. Mol. Spectrosc.* **290**, 13–17, 10.1016/j.jms.2013.06.004 (2013).

[181] Amat, G. & Pimbert, M. On Fermi resonance in carbon dioxide. *J. Mol. Spectrosc.* **16**, 278–290, 10.1016/0022-2852(65)90123-2 (1965).

[182] Rothman, L. S. & Young, L. D. G. Infrared energy levels and intensities of carbon dioxide–II. *J. Quant. Spectrosc. Radiat. Transf.* **25**, 505–524, 10.1016/0022-4073(81)90026-1 (1981).

[183] Toth, R. A., Brown, L. R., Miller, C. E., Devi, V. M. & Benner, D. C. Spectroscopic database of CO_2_ line parameters: 4300 − 7000 cm^−1^. *J. Quant. Spectrosc. Radiat. Transf.* **109**, 906–921, 10.1016/j.jqsrt.2007.12.004 (2008).

[184] Furtenbacher, T. 626M24 dataset of ^12^C^16^O_2_10.17605/OSF.IO/9PZEW (2024).

